# The contribution of non-coding regulatory elements to cardiovascular disease

**DOI:** 10.1098/rsob.200088

**Published:** 2020-07-01

**Authors:** Diego Villar, Stephanie Frost, Panos Deloukas, Andrew Tinker

**Affiliations:** 1 Blizard Institute, Barts and the London School of Medicine and Dentistry, Queen Mary University of London, 4 Newark Street, London E1 2AT, UK; 2 William Harvey Research Institute, Heart Centre, Barts and the London School of Medicine and Dentistry, Queen Mary University of London, Charterhouse Square, London EC1M 6BQ, UK

**Keywords:** cardiovascular, gene regulation, GWAS, epigenetics, functional genomics

## Abstract

Cardiovascular disease collectively accounts for a quarter of deaths worldwide. Genome-wide association studies across a range of cardiovascular traits and pathologies have highlighted the prevalence of common non-coding genetic variants within candidate loci. Here, we review genetic, epigenomic and molecular approaches to investigate the contribution of non-coding regulatory elements in cardiovascular biology. We then discuss recent insights on the emerging role of non-coding variation in predisposition to cardiovascular disease, with a focus on novel mechanistic examples from functional genomics studies. Lastly, we consider the clinical significance of these findings at present, and some of the current challenges facing the field.

## 1. Introduction

Human individuals differ from each other in millions of DNA sequence variants, some of which contribute to phenotypic differences in physiology and disease predisposition. Cardiovascular disease (CVD) comprises a range of monogenic and complex diseases accounting for a quarter of deaths worldwide. The study of family pedigrees has revealed new molecular players in monogenic cardiovascular disorders such as familial forms of cardiomyopathy, yet the most prevalent cardiovascular conditions are complex diseases with a heritable component. Facilitated by technological advances in sequencing technology, genome-wide association studies (GWAS) have analysed human genetic variation across individuals presenting a range of cardiovascular traits and diseases [1–3]. These comprise physiological parameters of cardiovascular function, such as heart rate or electrocardiogram measurements, and common pathologies such as cardiac arrhythmias and coronary artery disease (CAD).

A general finding from cardiovascular GWAS is the high prevalence of common genetic associations in the non-coding fraction of the human genome. The majority of human loci associated with cardiovascular traits and diseases are not in linkage disequilibrium (LD) with coding exons [4], thus a large fraction of genetic associations must reflect the effect of variation in gene regulation. Here, we will review recent evidence from genetic studies into the potential role of non-coding DNA elements in cardiovascular physiology and disease, with discussion of mechanistic insights from molecular approaches evaluating the effect of non-coding genetic variation on gene regulation and cellular phenotypes.

## 2. Genetic association studies in cardiovascular disease

**Figure 1.**
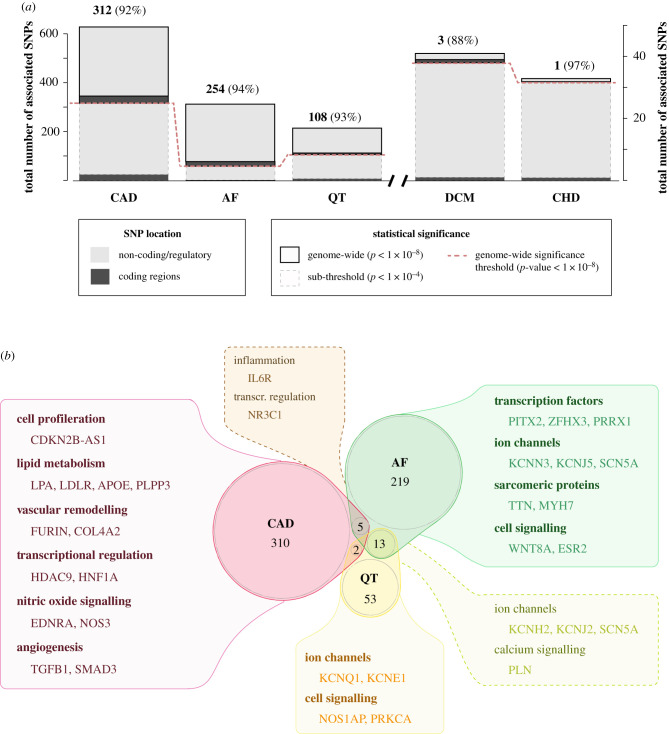
Summary of genome-wide association signals and proximal genes for major cardiovascular diseases and traits. (*a*) Total number of associated single nucleotide polymorphisms (SNPs) for coronary artery disease (CAD), atrial fibrillation (AF), QT interval duration (QT), idiopathic dilated cardiomyopathy (DCM) and congenital heart disease (CHD). For each disease or trait, top solid-outline boxes correspond to SNPs at genome-wide significance with *p*-value lower than 1 × 10^−8^, and bottom dashed-outline boxes to sub-threshold SNPs (*p*-value < 1 × 10^−4^). The dashed red line denotes the genome-wide significance threshold. SNPs located in coding regions of the genome are represented as black bars, and with those in non-coding segments in light grey. Numbers on top of each bar indicate the total number of associated SNPs at a genome-wide level of significance (*p*-value < 1 × 10^−8^), and percentages the non-coding fraction across all associated SNPs. Data from [5]. (*b*) Genes proximal to the associated SNPs in (*a*). (*p*-value < 1 × 10^−8^) are represented as a Venn diagram for CAD, AF and QT (centre). Numbers indicate gene counts in each region of the plot, including genes associated with two traits/diseases. The boxes linked to each region summarize example genes proximal to GWAS signals, and the functional categories they belong to.

Population genetics in CVD has classically focused on the analysis of familial pedigrees of suspected monogenic disorders, and for the last decade on association studies investigating complex diseases genome-wide. For common traits and diseases, GWAS are typically conducted on hundreds to thousands of unrelated individuals that are genotyped on up to two million single nucleotide polymorphisms (SNPs) found in the population at high frequency (greater than 5% for minor alleles). Genotyped SNPs are then used to impute between 10 and 20 million additional variants including indels, and each of these variants is tested for association with a trait of interest, providing a global view of potential candidate disease loci across the whole genome. Recently, SNP arrays have been complemented by including low-frequency (MAF 1–5%) and rare (MAF < 1%) non-synonymous coding variants. In this section, we discuss the findings of GWAS across key types of CVD, and their relevance to our understanding of how non-coding regions of the genome may contribute to disease risk (see also figure 1).

Association signals from GWAS are commonly reported for variants showing a *p*-value lower than 1 × 10^−8^, which is widely accepted as a genome-wide significance threshold (figure 1*a*, top bars with solid outline). More recently, several studies have also interrogated association signals at higher *p*-values (typically *p* < 1 × 10^−4^), under the assumption that these may also include functionally relevant signals (figure 1*a*, bars with dash outline). Sub-threshold variants have been shown to include those at a false discovery rate lower than 5% [6], and to enrich for tissue-specific epigenetic signals [7,8]. Nevertheless, it is likely they are overall weaker in their functional significance when compared to variants at genome-wide significance, as suggested by their modest contribution to genetic risk scores [6]. For clarity, we focus our discussion of GWAS findings on reported variants at genome-wide significance (*p* < 1 × 10^−8^), which to date account for most of those that have been functionally characterized.

### 2.1. Coronary artery disease and myocardial infarction

CAD due to atherosclerosis and potential myocardial infarction (MI), after occlusion of a coronary artery due to plaque rupture, is a major cause of mortality and morbidity. CAD is a paradigm of complex disease, with an estimated heritability of 50% [9] and multiple risk factors (e.g. high lipid levels, hypertension, diabetes and inflammation) which in turn appear to be under genetic control. Given its huge societal burden, CAD and MI were among the first complex diseases to be targeted in GWAS.

To date, genetic associations studies have collectively assessed over 70 000 CAD cases and hundreds of thousands of controls, revealing over 160 genomic loci associated with CAD and MI at a genome-wide level of significance (reviewed in [10]). CAD and MI share a very similar genetic architecture, with two notable exceptions (the *Abo* and the *Hdca9* loci) [11]. In line with the complex nature of CAD, genetically associated loci are involved in a range of biological processes, such as lipid metabolism, vascular tone, inflammation or vascular remodelling [6,12]. However, despite important biological insights into variants regulating cholesterol metabolism [13,14] and nitric oxide signalling [15], the precise molecular mechanisms underlying the majority of associated SNPs with CAD risk are largely unknown.

The complex genetic association data in CAD also exemplifies the importance of co-occurrence of common risk variants in a given individual, and their potential joint contribution to disease risk. Given the number and population frequency of genetic variants associated with CAD, an individual is expected to carry over a hundred of currently known risk alleles. In this regard, a recent evaluation of polygenic scores [16] estimated 8% of the population being at more than threefold increased risk for CAD—a prevalence 20 times higher than the carrier frequency of rare monogenic mutations conferring a comparable disease risk [17].

### 2.2. Congenital heart disease

Congenital heart disease (CHD) is the most common type of birth defect (affecting approx. 1% of live births) and comprises structural abnormalities affecting the heart septum, valves and the outflow tract. CHD is genetically heterogeneous, and even within the same abnormality, differences in clinical symptoms across patients are very common [18]. The post-natal survival of CHD patients has dramatically improved in the last decade due to the implementation of corrective cardiac surgery. However, most CHD patients suffer cardiac complications in adulthood, with arrhythmias and heart failure being the most common.

Some forms of CHD are associated with well-known chromosomal abnormalities (such as trisomy of chromosome 21 [19]) and copy number variations, which account for approximately 25% of cases [20]. Studies to date have identified over 50 genes with point mutations associated with familial CHD, which largely correspond to cardiac developmental genes (e.g. transcription factors *GATA4* and *NKX2–5*). However, for the vast majority of CHD patients, no apparent chromosomal abnormality or causal mutation can be identified. In fact, familial studies have often failed to identify a single candidate genetic variant that completely segregates with the disease [21,22]. These findings collectively suggested a significant contribution of *de novo* mutations and polygenic inheritance in CHD.

Because congenital defects are expected to be under strong selective pressure, *de novo* mutations may account for a significant fraction of sporadic CHD occurrences. Recent studies analysing hundreds of disease cases and controls have shown an increased burden of *de novo* mutations in genes involved in transcriptional regulation, including enzymes controlling the reading and removal of histone 3 lysine 4 methylation [23,24] and the pleiotropic transcription factor *NR2F2* [25]. *De novo* mutations may account for up to 10% of CHD cases [26], with an increased prevalence among patients where CHD is accompanied by extra-cardiac abnormalities such as neurodevelopmental disorders [24]. More recently, exome sequencing of approximately 2500 trios suggested distinct gene pathways associated with *de novo* and recessive forms of CHD, with mutations in chromatin-modifying genes being enriched in *de novo* sporadic cases [27].

The potential role of polygenic inheritance in CHD has been highlighted by recent GWAS studies focused on common genetic variation, which to date have identified a relatively low number of associations at a genome-wide level of significance (figure 1*a*). Two strong associations were identified in tetralogy of Fallot patients, one of which falls in a large intronic region with potential regulatory function [28]. Across thousands of CHD cases encompassing all major clinical types, genetic associations were most significant when patients were segregated by CHD subtype, and the strongest association was found in an intergenic region proximal to the homeobox transcription factor *MSX1* [29].

These studies, therefore, suggest a key role for gene regulatory processes in mediating congenital heart disease. Focusing on regulatory regions of the developmental transcription factor *TBX5*, Smemo *et al*. [30] were among the first to provide molecular and mechanistic evidence for the role of transcriptional enhancers in CHD—more recent examples will be the focus of later sections of this review.

### 2.3. Long QT syndrome

The continuous and coordinated contraction of the heart relies on tightly regulated opening and closing of specialized ion channels in heart muscle cells. Cardiac arrhythmias result from disruptions in myocyte electrical function and are a leading cause of sudden cardiac death, stroke and heart failure. The genetics of a wide spectrum of cardiac rhythm disorders has been reviewed elsewhere [31]—here, we will focus on two common arrhythmias, namely long QT syndrome (LQTS) and atrial fibrillation (AF), which have been the focus of recent genetic studies.

Cardiac arrhythmias have been classically studied within family pedigrees, such as in the pioneering work that led to the mapping of the chromosomal loci underlying hereditary LQTS [32]. This form of inherited arrhythmia leads to a prolonged duration of ventricular action potentials, and thus an extended QT interval on the electrocardiogram. Affected individuals have a propensity to a particular form of ventricular tachycardia known as torsade-de-pointes, and this can degenerate into ventricular fibrillation leading to sudden death if untreated. Familial studies identified three major causative genes in two potassium and the cardiac sodium channel genes, which gave rise to the classification of patients into three main LQTS types. Later work extended the number of genes linked to hereditary LQTS to 15, although most of these additional mutations are very rare (reviewed in [33]). In most cases, LQTS mutations lead to defective channel function or trafficking, as shown in a number of experimental systems—including engineered mouse models [34] and human patient-derived induced pluripotent stem cells (iPSCs) [35].

The QT interval is heritable in the wider population and GWAS have identified common genetic variants associated with this electrocardiographic trait. The first study [36] identified a strong association signal in the *NOS1AP* locus, a potential regulator of ion channel function [37]. Subsequent studies have identified genetic variants associated with electrocardiographic traits [38,39]. These are proximal to many of the same ion channel genes that harbour familial LQTS mutations, in addition to some developmental transcription factors (e.g. *MEIS1* and *TBX5*) [40]. A recent GWAS of QT interval duration across over 70 000 individuals identified loci associated with cardiomyocyte calcium handling, highlighting a potential role of repolarization pathways in LQTS susceptibility [41] (see also figure 1).

The variable clinical manifestations in LQTS patients with the same gene mutations [42] have led to the investigation of potential modifier genes contributing to incomplete penetrance or variable expressivity (reviewed in [43])—including non-coding genetic variants). An elegant study focusing on a South African founder population for the KCNQ1-A341 V mutation [44] demonstrated that two common non-coding *NOS1AP* variants associated with increased risk of life-threatening arrhythmia in LQTS patients. In fact, variants in this locus have also been linked to drug-induced LQTS risk [45]. Conversely, a case-control study in LQTS duos identified an intronic *KCNQ1* variant associated with lower arrhythmic risk [46]. Recently, iPSCs were generated from several members of a family with type 2 LQTS, including severely and mildly affected individuals [47]. By combining exome sequencing and electrophysiological measurements, the authors revealed a protective gain-of-function mutation in *KCNK17* and an aggravating variant in *REM2*. In sum, these studies have provided proof-of-principle evidence for the existence of predisposing and protective modifier genes in LQTS. Recent prospective analyses assessing the value of multi-locus genetic scores [48] illustrate the potential for variants involved in disease predisposition to inform clinical management of LQTS patients.

### 2.4. Atrial fibrillation

AF is the most common cardiac arrhythmia (affecting up to 2% of the general population). It occurs due to abnormal electrical impulses firing in the atria that override the natural pacemaker of the heart, and lead to an uncontrolled and highly irregular heart rhythm. Although AF can develop secondarily to chronic cardiac stress and other heart diseases, it has a substantial genetic heritability estimated at approximately 60% [49]. Initial investigations into the genetic causes of AF focused on candidate gene screening in cohorts of early-onset lone AF patients or families showing Mendelian disease segregation. Although these classical studies discovered a number of rare coding variants in ion channel genes responsible for generating the atrial action potential [50], recent population-based approaches suggest coding mutations account for a relatively small proportion of genetically determined AF [51].

By contrast, analyses of common and rare variation across AF patients and controls suggest an important role for non-coding genetic variants in mediating disease susceptibility. The first major AF GWAS identified a significantly associated locus in an intergenic non-coding region on chromosome 4q25, adjacent to the *PITX2* gene [52]. The AFGen Consortium [53] and subsequent meta-analyses [54,55] have extended these GWAS to hundreds of thousands of AF patients and controls. To date, these efforts have collectively identified over 100 genomic regions associated with AF, the majority of which lie within introns or intergenic regions.

Genetic loci associated with AF are often within the non-coding regions of ion channel genes such as *KCNQ1*. These variants may predispose to AF by shortening or lengthening atrial action potential leading to changes in atrial tissue refractoriness, and can be carried in multiple combinations of rare and common variants with epistatic effects [56]. The second set of associated GWAS loci are proximal to transcription factors such as *PITX2*, *ZFHX3*, *GATA4*, *NKX2–5* and *TBX5*—mostly with previously known roles in heart development. Four AF-associated regions at 4q25 are closely associated with the *PITX2* gene, and thus deficiency of the major heart isoform *PITX2c* was thought to mechanistically contribute to AF. In agreement, haploinsufficient *PITX2* mice show a propensity to atrial arrhythmias and AF [57], albeit coding mutations in *PITX2* are uncommon in AF patients [58]. The role of non-coding SNPs corresponding to the four association signals in the 4q25 locus is still under investigation, and recent studies have demonstrated the interaction of enhancer elements in this region with *PITX2*, with significant differences in *PITX2c* expression levels between allele variants [59]. Intriguingly, evaluation of *PITX2c* expression in human AF patients and controls have been inconclusive in observing significant differences [60], suggesting the effects of PITX2 on AF susceptibility may occur during development or in response to cardiac stress in the adult. In this regard, PITX2 is known to interact with other transcription factors whose loci also harbour AF-associated non-coding variants (e.g. ZFHX3 and TBX5)—suggesting an altered atrial regulatory network may play a role in AF pathogenesis (reviewed in [61]). Thirdly, GWAS loci associated with AF are also found close to structural heart muscle genes, such as those encoding for sarcomeric MYH6/7, TTN and associated regulatory proteins (e.g. CAMK2D). Variants in these loci potentially contribute to an atrial cardiomyopathy that may be exacerbated by chronic cardiac stress. In this regard, MYH7 expression has been shown to be elevated in human patients and animal models of chronic AF [62].

Despite the plethora of genetic associations in AF patients (figure 1), genetic testing is not recommended in standard clinical diagnosis. The complexity of GWAS associated genes in AF and a limited knowledge on the causative role of most candidate genes probably underlie the poor performance of polygenic scores based on GWAS [63].

In sum, genetic studies suggest a role for transcriptional enhancers and non-coding genetic variation in contributing to disease penetrance in LQTSs, as well as for regulatory networks of cardiac development in mediating onset and progression of AF. Mechanistic examples of specific loci will be discussed in §4 of this review.

### 2.5. Cardiomyopathies

Cardiomyopathies are a heterogeneous group of diseases characterized by impairment of heart muscle function. Although they can be secondary to factors such as ischaemia or hypertension, cardiomyopathies are often heritable. Although left ventricular hypertrophy and systolic heart failure often associate with cardiomyopathies, we will not cover either here due to their very diverse aetiologies [64]. Instead, here we focus on the two major clinical types of primary cardiomyopathies: hypertrophic cardiomyopathy (HCM) and dilated cardiomyopathy (DCM). HCM is a major cause of sudden death and heart failure, and functionally defined by a characteristic pattern of ventricular hypertrophy and diastolic dysfunction; whereas DCM manifests with ventricular dilation and systolic dysfunction.

More than half of HCM patients have a family history of the disease, with a 20–35% figure in DCM. Therefore, genetic studies have classically focused on linkage analysis and candidate gene approaches, revealing rare variants associated with familial cardiomyopathy. HCM is mainly linked to mutations in genes for sarcomeric proteins, especially MYH7 [65,66]. Linkage analysis in HCM families not linked to the MYH7 locus has identified mutations in additional genes encoding contractile elements of the sarcomere, such as MYCBP3 or TTN2. Further sequencing of candidate loci found HCM-associated mutations in genes for other components of the contractile machinery, including MYL2, MYL3 and TNNC1. Overall, contractile element mutations are found in nearly half of patients with familial HCM, leading to the notion that this cardiomyopathy is primarily a disease of the cardiac sarcomere [67].

By contrast, DCM patients display substantial genetic heterogeneity. More than 50 genes have been linked to familial DCM, some of which overlap with those associated with HCM. A major DCM-associated gene is *TTN*, encoding for the giant protein Titin—mutations in *TTN* are associated with 20% of DCM cases [68]. However, current genetic testing of DCM patients, even with large panels of genes, has only approximately 50% sensitivity in detecting mutations. Even within the same family, there is substantial heterogeneity of symptoms for individuals carrying the same rare variant. Moreover, the frequency of known pathogenic mutations in HCM and DCM is much lower than the prevalence of these diseases, suggesting up to 3–5% of the population carries genetic variants associated with cardiomyopathy (i.e. a significant fraction of patients have no characterized pathogenic variants). In line with these findings, recent large-scale exome sequencing of DCM patients suggested a robust disease association for only 12 of 56 genes commonly implicated in DCM [69].

GWAS studies of cardiomyopathy patients have detected few associated non-coding variants at a high level of significance (figure 1*a*). GWAS of DCM [70,71] identified a number of susceptibility loci, corresponding to non-coding variants proximal to *HLA-C* and *HSP7*, and common coding variants in the *BAG3* gene, for which rare variants were also found in familial DCM [72]. More recent studies in specific populations have reported non-coding associated variants in the *CACNB4* cardiac channel locus [73] (in an African American population), and rare coding variants in the developmental cardiac TF *NKX2–5* and the cytoskeletal protein *FLNC* in Icelanders [74]. Overall, the low number of associated loci identified may be related to idiopathic DCM often being a consequence of pre-existing conditions (such as congenital heart disease). It is therefore possible that genetic heterogeneity of DCM GWAS cohorts is substantially higher than that of other CVD GWAS, as suggested by the low replicability of association signals across studies.

The above results also contrast with reports quantifying the degree of gene expression changes in DCM patients, which have documented over 250 genes as differentially expressed [75]. Among these were many DCM-associated genes in GWAS, including the cardiac transcription factor TBX20 [76] and many of its target genes. Focusing on a carefully phenotyped cohort, a recent study has also revealed structural genetic variants (e.g. insertions, deletions and duplications) associated with DCM [77], most of which reside in non-coding regions. Nevertheless, the mechanistic role of the few non-coding genetic variants associated with cardiomyopathies remains poorly understood.

## 3. Experimental approaches for functional annotation of non-coding variation

By design, the GWAS studies discussed above identify broad genomic regions (loci) associated with CVD, which are often proximal to several candidate genes. This is because common genetic variants in the human genome are pervasive (occurring on average every 300 bp) and typically co-segregate in blocks of genomic DNA. This co-segregation of alleles is termed LD, and is often the case that GWAS lead variants will be in high LD (*r*^2^ > 0.8) with many other sequence variants within a locus. Moreover, comprehensive catalogues of common sequence variation such as the 1000 Genomes Project allow consideration of all candidate variants. These factors complicate the identification of the individual variant(s) underlying a GWAS association signal, and have prompted the development of statistical methods to identify credible subsets more likely to contain causal variants (reviewed in [78]).

In parallel, functional interpretation of GWAS results has prompted detailed and careful annotation of association intervals, spanning variants in high LD with a lead variant and sub-threshold genomic variants. In this section, we will review some of the current experimental approaches aimed at the functional annotation of GWAS variants and associated loci, with a focus on epigenomic approaches and the complementary evidence they provide for an investigation of non-coding regions associated with CVD. There are two related areas we will not cover here. First, *in vivo* transgenic reporter assays provide rich information on the tissue and developmental specificity of candidate regulatory elements, and are often used in combination with the strategies we discuss [79]. Second, we will not discuss gene expression annotation of GWAS in detail, such as the use of expression quantitative trait loci (eQTLs) for variant interpretation. A substantial fraction of non-coding GWAS signals corresponds to eQTLs, and their use for the annotation of cardiovascular GWAS has been reviewed elsewhere [80].

### 3.1. Epigenetic annotation of non-coding elements

When combined with specific experimental protocols, sequencing technologies enable genome-wide profiling of hallmarks of regulatory activity across the genome. Transcription factor binding can be assessed genome-wide to identify all genomic elements bound by a protein in a tissue or cell line of interest, using chromatin immunoprecipitation coupled to DNA sequencing (ChIP-seq). This strategy can also be applied to histone modifications to identify DNA regions harbouring epigenetic marks characteristic of active or inactive regulatory elements. By leveraging these approaches, international consortia such as the ENCODE and Roadmap Epigenomics have discovered the unexpectedly high proportion of human non-coding DNA containing biochemical signatures of regulatory activity [81]. These efforts have profiled the binding of over a hundred transcription factors in human cells and tissues, as well as enrichment of histone modifications associated with the regulatory activity (e.g. promoters and enhancers). Their results suggest over 35% of the human genome can be considered as functional based on its potential to regulate tissue-specific expression patterns. Moreover, similar genome-wide approaches to profile RNA transcripts have indicated the human genome is also pervasively transcribed [81].

These genome-wide profiles provide invaluable information to interrogate genomic regions from GWAS, for example, to examine whether associated variants localize within regions with regulatory activity in a disease-relevant tissue. In fact, a recent epigenomic analysis of over a hundred human samples encompassing major tissue types [82] has highlighted the tissue-specific nature of a good fraction of genetic association signals. Non-coding GWAS signals were strongly enriched among genomic elements harbouring marks of regulatory activity, most notably H3K4me1 and H3K27ac (both characteristic of enhancer elements). This enrichment was markedly tissue-specific: across CVDs and traits, GWAS signals were most enriched in heart, vasculature and liver epigenomes. These results highlight the importance of investigating GWAS genomic regions in relevant tissues and cellular models, and in the context of dynamic regulatory environments [83]. Of note, recent work from the mouse ENCODE consortium reported significant conservation of human and mouse regulatory landscapes [84], supporting the utility of comparing human and mouse epigenome annotations.

**Figure 2.**
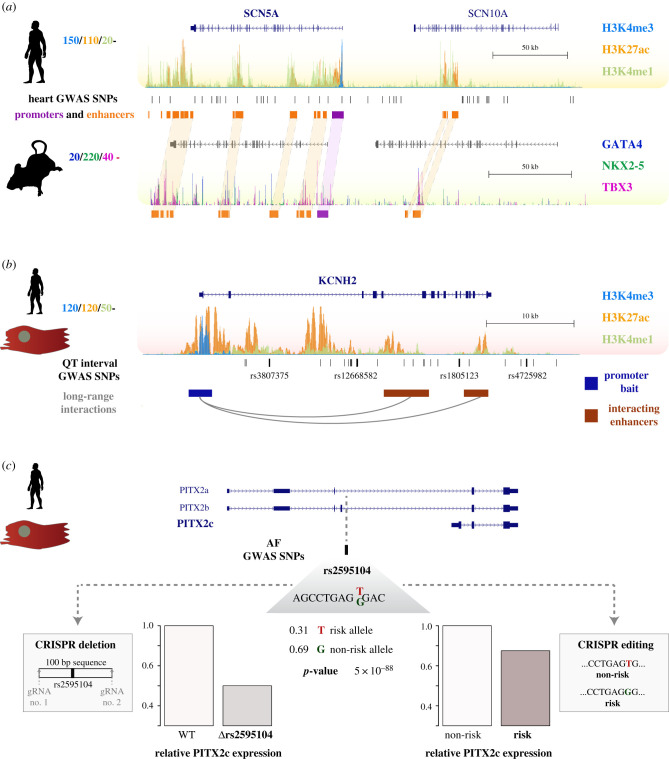
Experimental approaches for functional investigation of non-coding elements associated with cardiovascular disease. (*a*) Epigenomic annotation of the *SCN5A/SCN10A* cardiac disease locus in human and mouse left ventricle. Human genome tracks show epigenome signals for three histone marks associated with regulatory activity (H3K4me3 in blue; H3K27ac in orange and H3K4me1 in green) [82]; GWAS lead SNPs in this locus (black bars); and putative promoters (purple) and enhancers (orange). Mouse genome tracks below show ChIP-seq data for cardiac transcription factors GATA4 (blue), NKX2–5 (green) and TBX3 (purple) [88]. Orthologous promoters and enhancers in the human and mouse loci are connected by light purple and orange guides, respectively. (*b*) Epigenomic annotation of the *KCNH2* QT interval locus in the human left ventricle (epigenome signals as in (*a*)). Bottom tracks show genetic variants associated with QT interval duration (black bars), and long range interactions between the *KCNH2* promoter (blue) and enhancer elements (red) [89]. (*c*) Genomic location of the lead genetic variant rs2595104, associated with atrial fibrillation (AF) and located upstream of the *PITX2c* transcript annotation. The grey inset shows the sequence context of the variant, the minor risk allele (0.31 frequency), the major protective allele (0.69 frequency) and the *p*-value of the AF association. The regulatory effect of this variant on *PITX2c* expression was analysed in iPSC-derived cardiomyocytes by CRISPR/Cas9 deletion of a 100 bp sequence encompassing rs2595104 (left barplot, 54% reduction); and also by CRISPR genetic editing producing isogenic cardiomyocytes carrying the major non-risk allele and the minor risk allele (right barplot, 27% difference). Adapted with permission from [59].

In this regard, recent work has begun dissecting genome-wide changes in epigenetic marks, transcription factor binding and chromatin accessibility during cardiac development and in response to stress (reviewed in [85]). Applying functional genomics to null mouse models for the cardiogenic transcription factors NKX2–5 and TBX5, Luna-Zurita *et al.* [86] documented extensive cooperativity between these transcription factors and GATA4, which could often be explained by specific binding motif arrangements. Moreover, genetic ablation of either factor alone or in double knock-outs showed interdependent binding between the three factors, including significant ectopic binding of the remaining two TFs upon ablation of either one—suggesting mutations in these factors found in CHD patients can act both by loss of TF binding and abnormal ectopic binding of cooperating TFs. Using a similar approach in an inducible TBX3 transgenic mouse, van den Boogard *et al*. [87] mapped genome-wide binding of the transcription factors TBX3, NKX2–5 and GATA4 in the developing heart, also documenting significant co-binding by the three TFs. Comparing gene expression changes upon TBX3 induction with regions bound by TBX3, the authors defined a core-set of ten ion channel genes as TBX3 targets, including the *SCN5A* cardiac sodium channel locus. Using their TBX3 ChIP-seq data, the authors identified candidate TBX3-bound enhancers in this locus and evaluated the heart-specific activity of orthologous human sequences in a mouse reporter assay. One of the functionally conserved human enhancers contained a SNP associated with cardiac arrhythmias, directly overlapping a TBX3 ChIP-seq peak and corresponding to a highly conserved position in the T-box binding consensus sequence. Accordingly, a reporter construct with the minor SNP allele did not respond to TBX3 overexpression, and associated with reduced activity by 60% in a zebrafish reporter assay. These two reports exemplify the power of epigenetic annotation approaches to inform transcriptional mechanisms in CVD, and the function of disease-associated non-coding variants (see also figure 2*a*).

Cardiac stressors such as mechanical overload play a well-documented role in heart disease. Under pressure overload, the heart has to contract and eject blood against excessive pressure, for example, caused by systemic hypertension or aortic stenosis. Over time, this form of stress results in pathological hypertrophy of the heart muscle, a process in which transcription factors active in the fetal heart are thought to be re-activated [90]. Several recent studies have employed functional genomics approaches to assess how extensive a ‘fetal gene programme’ is in cardiac hypertrophy. He *et al*. [91] focused on transgenic and knock-out *GATA4* mouse models to investigate this cardiac TF in mouse fetal and adult hearts, and in response to pressure overload. Their results showed GATA4 binding differed markedly between fetal and adult samples: while two-thirds of adult GATA4-bound regions were also bound in E12.5 mouse hearts, 80% of fetal GATA4 peaks were not bound in the adult. Comparison with H3K27ac data and genes regulated downstream of GATA4 indicated an important functional role for regions showing both GATA4 binding and H3K27ac signal, suggesting binding of this transcription factor mediates H3K27ac deposition and subsequent transcriptional changes. Upon pressure overload in adult mice, upregulated genes were significantly associated with fetal-specific GATA4-bound regions. However, cardiac stress-induced GATA4 binding across a much larger set of new, stress-specific genomic regions. An earlier study [92] also found widespread changes in gene expression and the level of activating (e.g. H3K27ac) and repressive histone modifications under pressure overload. More recently, histone modifications associated with active transcriptional enhancers and downstream gene expression were profiled in left ventricle tissue from 18 idiopathic DCM patients and healthy controls [93]. Analyses of these data found over 7000 disease-associated regions showing gains or losses of regulatory activity. By comparison with data from the human fetal heart, the authors found around half of the disease-associated regions shifted towards a fetal-like epigenome and transcriptome. While consistent with the reactivation of fetal enhancers under cardiac stress and disease, these results also suggest a significant activation of new stress-responsive enhancers—which are not active during cardiac development but may also contribute to heart disease [94].

### 3.2. Long-range chromatin interactions

A key property of non-coding regulatory elements such as transcriptional enhancers is long-range gene regulation: target genes of regulatory elements can be located hundreds of kilobases away in the linear DNA sequence, complicating efforts to assign effector genes of disease-associated genetic variants located in the non-coding genome. However, a range of experimental approaches can be used to detect interactions between transcriptional enhancers and the promoter regions of distant genes—thereby addressing an important limitation of the epigenetic annotation methods discussed above. The most common of these techniques are *in situ* fluorescent hybridization (FISH) or experiments based on proximity ligation of cross-linked chromatin fragments (known as chromosome conformation capture) [95]. Although both techniques have specific advantages and disadvantages, FISH is intrinsically a low-throughput approach, and typically favoured to study specific loci and interactions, and to independently validate findings from proximity ligation methods (see [96] for a detailed discussion).

We focus below on chromosome conformation capture approaches, and their application in the cardiovascular area. A critical aspect in designing these experiments for the study of gene regulation is tissue-specificity, both for three-dimensional chromatin interactions and (especially) the activity of interacting regulatory elements. This factor underlies the importance of assaying the right cell type and developmental stage for meaningful analyses, as exemplified in the studies discussed in this section.

Some of the key genetic loci associated with cardiac disease have been analysed with chromosome conformation capture approaches. Natriuretic peptide A and B (encoded by the neighbouring *NPPA* and *NPPB* genes) become expressed in the atrial and ventricular myocardium during development, and are strongly induced in the ventricles upon cardiac stress and heart failure. Because of their paradigmatic roles in heart development and disease, their regulatory landscapes and three-dimensional organization were analysed with an adaptation of chromatin conformation capture (4C-seq) [97]—in which locus-specific interrogation of promoter–enhancer interactions is achieved by sequencing all DNA fragments interacting with a chosen genomic region. By choosing viewpoints on each side of the *NPPA/NPPB* locus, the authors showed three-dimensional interaction profiles that were largely maintained in the adult and fetal heart, as well as under cardiac stress induced by pressure overload. These interactions were even present in the liver, a tissue where the two genes are not expressed, suggesting the three-dimensional conformation of the locus is permissive, yet leads to gene expression specifically in the heart. Employing an elegant combination of the 4C-seq approach, H3K27ac ChIP-seq profiles and transgenic reporter assays, the study also found adult and fetal enhancers were largely distinct, with developmental enhancers being located more distally. However, and in line with results discussed in the previous section [91], stress-responsive enhancers for *NPPA/NPPB* were largely distinct from those regulating its developmental expression—thus pointing to specific regulatory mechanisms under cardiac stress. Similar locus-specific studies for the *SCN5A* and *KCNH2* cardiac arrythmia loci have combined profiling of regulatory landscapes and three-dimensional interactions with experimental perturbations [88,98]—these will be discussed in the next section (see also figure 2*a* and *b*).

More recently, chromatin conformation capture experiments have been adapted to interrogate three-dimensional DNA interactions across the whole genome, an approach termed Hi-C. Such chromatin interaction maps are incredibly complex, and interactions can be selected for specific genomic regions such as gene promoters. In an impressive study aimed at annotating non-coding genetic variants associated with CVD, Montefiori *et al*. [89] used this promoter–capture adaptation of Hi-C to map genomic interactions with the promoters of approximately 22 000 human genes. By applying this approach to human-iPSCs and derived cardiomyocytes (iPSC-CMs), this study provided a detailed comparison of three-dimensional genome architecture in a chief experimental system of heart muscle function. Their results revealed approximately 400 000 enhancer–promoter interactions in these cell types, over half of which were shared between iPSCs and their derived CMs. Leveraging matched H3K27ac and H3K4me1 data, the authors showed chromatin interactions specific to either iPSCs or CMs were enriched in regulatory elements with active epigenetic marks in the matched cell-type. Moreover, integration of these results with GWAS SNPs across a wide range of CVDs showed approximately 20% of LD SNPs located within distal genomic elements interacting with promoters of their likely target genes. In fact, more than 90% of SNP-target interactions did not involve the nearest gene, highlighting the importance of long-range regulation for functional interpretation of non-coding genetic variants. Of note, an earlier study employing an enhancer-centric Hi-C approach on human coronary artery smooth muscle cells (HCASMCs) [99] reached similar conclusions for GWAS variants associated with CAD—89% of SNP-promoter contacts in HCASMCs skipped at least one gene, and 64% were mapped to more than a single gene target. More recently, an integrative analysis of genetic variants associated with AF has also made use of epigenome, transcriptome and promoter-capture Hi-C data to score and prioritize genomic loci for functional validation [8].

### 3.3. Perturbation of non-coding regions by genome and epigenome editing

A classic limitation in functional genomics studies has been the reliance on correlative evidence to inform genome function. As such, the above approaches annotate putative regulatory elements based on their epigenetic annotation (§3.1), and their long-range interactions across the genome (§3.2). However, these strategies do not address the impact of candidate promoters and enhancers (and the genetic variants they harbour) in cellular and organismal functions. Thanks to major developments in genome-editing technologies in the last decade, it is now becoming feasible to functionally perturb non-coding genomic elements. These technologies have been extensively reviewed recently [100], and here we will focus on illustrative applications of genome-editing applied to non-coding elements in the cardiovascular area. A good fraction of such recent work employs the CRISPR/Cas9 technique, an approach based on the expression of an RNA-directed and sequence-specific bacterial nuclease, which can be employed for genetic or epigenetic editing of specific non-coding elements and genetic variants. However, classical genetics based on homologous recombination has also been successfully employed to target regulatory elements [101].

Recent work by Dickel *et al.* [102] provided a powerful example of genetic deletion of candidate genomic elements associated with CVD. Leveraging a range of epigenetic datasets in adult and fetal heart tissue from mouse and humans, the authors built a compendium of approximately 10 000 genomic elements with evidence of enhancer function in the heart [102]. By comparing these regions with current catalogues of human genetic variants associated with cardiac traits and diseases [5], Dickel *et al.* [102] found approximately 900 variants in high-scoring heart enhancers. In elegant proof-of-principle experiments, genetic deletion of two predicted enhancers proximal to the sarcomeric genes *MYL2* and *MYH7* led to measurable cardiomyopathy phenotypes in the mouse, such as cardiomyocyte disarray and reduced cardiac output. Overall, these experiments provide compelling evidence for the functional impact of GWAS variants in cardiac disease phenotypes *in vivo*. More recently, a similar approach was taken to delete large genomic regions encompassing non-coding genetic variants associated with AF [8].

*In vitro* genetic editing in human models represents an exciting complementary approach to investigate the function of disease-associated variants and the genomic regions they occur in. For example, iPSC models of cardiomyocyte function can be used to interrogate non-coding regions associated with myocardial pathologies in which marker gene expression substantially differs between humans and mice [103]. A recent study focusing on the p.R453C-*MYH7* pathogenic mutation in HCM [104] highlights the usefulness of these models for detailed characterization of myocardial phenotypes associated with discrete genetic perturbations. By careful CRISPR/Cas9 engineering of this mutation across three independent iPSC lines (for wild-type, heterozygous and homozygous mutants), the authors demonstrate the dose-dependent contribution of this coding mutation to several hallmarks of HCM, such as increased cardiomyocyte size, sarcomeric disarray and arrhythmogenic alterations in calcium handling. This study illustrates the potential of human *in vitro* models for functional investigation of non-coding disease-associated variants and the regulatory elements they reside in—recent studies in this direction are discussed in the next section (see also figure 2*c*).

CRISPR/Cas9 approaches have also been adapted to modify the epigenome, although their application to cardiovascular loci has been limited to date. By combining a dead Cas9 protein with transcriptional activators or repressors, CRISPR activation and CRISPR inhibition (CRISPRi) approaches allow targeted perturbation of regulatory elements without direct genetic modification. A recent high-throughput study used CRISPRi for functional perturbation of regulatory epigenomes [105]. The study used an elegant approach with CRISPRi of a gene of interest in conjunction with FISH labelling of single cells (according to their RNA expression for the targeted gene). By flow-sorting perturbed cell populations based on the RNA FISH signal, the method allows the binning of cells according to their expression level for the gene of interest, which is associated with abundance of guide RNAs in that bin by high-throughput sequencing. The authors applied this strategy to the analyses of over 3000 enhancer-gene connections across 30 genes—including complex connections involving enhancers regulating up to five genes, and individual genes regulated by up to 14 distal elements. Consistent with the high degree of redundancy and robustness in mammalian regulatory landscapes, a relatively small 141 of the tested interactions between regulatory elements and genes led to significant effects on gene expression, which were of modest effect size on average (median 22%).

## 4. Mechanistic insights into the role of non-coding regulatory variation in cardiovascular disease

This section will focus on selected examples of the application of complementary experimental approaches (§3) to the investigation of genetic regions associated with CVD, with a focus on non-coding variants and the larger promoter and enhancer elements they occur in. For completeness, we summarize selected and additional studies in table 1. Finally, we discuss the mechanistic insights these studies are starting to provide on the role of regulatory elements in diseases of the cardiovascular system.

**Table 1.** Summary of experimental studies of regulatory elements and trait-associated variants in cardiovascular disease. Abbreviations: ECs, endothelial cells; SMCs, smooth muscle cells; VSMCs, vascular smooth muscle cells; iPSCs, induced pluripotent stem cells; iPSC-CMs, cardiomyocytes derived from induced pluripotent stem cells; ESC-CMs, cardiomyocytes derived from embryonic stem cells; HCFs, human cardiac fibroblasts.

category	species	model	locus	variant(s)	experimental perturbation	experimental evidence	ref.
***coronary artery disease***							
non-coding interval	mouse	whole organism	*CDKN2A/B*	rs12555547 rs10757274 rs2383206 rs10965244	homozygous deletion	increased mortality reduced CDKN2A/B expression increased proliferation/reduced senescence in VSMCs	[101]
distal enhancer	human	LCLs HUVECs	*CDKN2B-AS1*	rs10811656 rs10757278	siRNA/natural genetic variation	STAT1 binding disrupted by risk haplotype long-range interaction between enhancer and CDKN2A/B locus STAT1 inhibits CDKN2BAS expression	[106]
non-coding interval	human	VSMCs	*CDKN2A/B* *CDKN2B-AS1*	rs1333049	natural genetic variation	risk genotype associated with reduced gene expression and increased proliferation	[107]
intronic interval	human	coronary artery ECs	*PHACTR1*	rs9349379	heterozygous deletion	MEF2 binding disrupted by risk allele 34 bp deletion leads to 35% reduction in PHACTR1 expression	[108]
intronic interval	human	coronary artery SMCs	*SMAD3*	rs17293632	natural genetic variation	SNP in open chromatin region, induced in TGFβ- or PDGF-BB-treated cells AP-1/TCF21 binding disrupted by protective minor allele	[109]
distal enhancer	human	iPSC-ECs and VSMCs	*PHACTR1*	rs9349379	90 bp enhancer deletion homozygous major/minor biallelic clones	enhancer deletion increased EDN1 expression minor risk allele associates with 20% increase in EDN1 expression	[110]
intronic enhancer	human	coronary artery ECs	*PLPP3*	rs17114036	enhancer deletion CRISPR inhibition	SNP in endothelial enhancer induced by haemodynamics enhancer deletion/repression leads to reduced PLPP3 expression KLF2 binding disrupted by risk allele	[111]
non-coding interval	human	iPSC-VSMCs	*CDKN2B-AS1*	risk/non-risk haplotype	60 kb deletion of risk and non-risk haplotypes	deletion of risk haplotype leads to non-risk like expression profile in iPSC-VSMCs	[112]
***congenital heart disease***							
distal enhancer	mouse	LacZ transgene	*TBX5*	rare variant	transgene expression	rare variant disrupts heart-specific expression and nuclear protein binding *in vitro*	[30]
intronic enhancer	human rat	heart tissue HEK293 cells H9C2 cells	*MTRR*	rs326119	natural genetic variation	minor allele disrupts CEBPA binding and associates with elevated plasma homocysteine levels	[113]
distal enhancer	human	normal iPSCs	*GATA4*	rs6601627 rs118065347	GATA4 small deletion by CRISPR	non-coding variants genetically associated with aortic bicuspid valve GATA4 deficiency impairs EMT in iPSCs	[114]
missense variant	mouse	knock-in mouse model	*PBX1*	*de novo* mutation	CRISPR genetic editing	multiple developmental defects in knock-in mice, including ventricular septal defects and persistent truncus arteriosus	[115]
***long QT syndrome***							
distal enhancer	human rat zebrafish	HL-1 cells zebrafish embryo RNVMs	*NOS1AP*	rs7539120	*in vitro* deletion NOS1AP overexpression	variant deletion reduces *in vitro* enhancer activity risk haplotype increases transgenic reporter activity and nuclear protein binding *in vitro*	[116]
missense variant	human	patient-specific iPSCs	*KCNH2*	novel missense variant	correction of variant in patient cells/homozygous knock-in in healthy cells	prolongation of action potential in patient iPSC-CMs and reduced *I*_Kr_. normalized phenotype in corrected cells	[117]
promoter region	human	human left ventricle HEK293 cells	*PRKCA*	rs9892651 rs9909004 rs7210446	natural genetic variation	minor protective allele associated with decreased PRKCA expression insert containing rs9909004 shows enhancer activity in HEK293 cells	[118]
***atrial fibrillation***							
distal enhancer	human	ESC-CMs	*PITX2c*	rs2595104	knock-in of risk allele approximately 100 bp enhancer deletion siRNA knock-down	30–54% reduced PITX2c expression upon SNP deletion; 27% reduction by risk allele effect dependent on TFAP2a/CITED2 preferential binding to non-risk allele	[59]
distal enhancer promoter/exon	mouse	whole organism	*GJA1* *KCNN3* *ZFHX3*	many in each genomic interval	40 kb homozygous deletion 85 kb homozygous deletion 33 kb homozygous deletion	reduced GJA1 expression in atria loss of KCNN3 expression in atria and ventricles no significant expression changes	[8]
distal enhancer	human	HL-1 cells HCFs iPSC-CMs	*PRRX1*	rs577676	PPRX1 gene knock-out	interaction between enhancer and PPRX1 promoter in cardiac fibroblasts variant required for enhancer activity PRRX1 suppression leads to shortening of atrial action potential	[119]
***Brugada syndrome***							
distal enhancer	mouse human	cardiac tissue	*SCN10A* *SCN5A*	rs6801957	BAC transgenic mouse	genetic variant interacts with SCN5A promoter engineered enhancer deletion essential for cardiac SCN5A expression risk allele associated with reduced enhancer activity	[88]
3′ UTR	human	blood samples	*SCN5A*	rs45592631	natural genetic variation	3′UTR variants identified in Tunisian family effect hypothesized to be dependent on miR-1270	[120]
***cardiomypathies***							
distal enhancers	mouse	whole organism	*MYL2* *MYH7*	proximal to coding mutations	homozygous deletions	cardiac abnormalities upon deletion of both enhancers (reduced fractional shortening, cardiomegaly and cardiomyocyte disarray)	[102]
missense variant	human	patient-specific iPSCs	*SCN5A*	R1898H	genetic editing to correct mutation	36% reduction in peak sodium current in patient iPSC-CMs	[121]
missense variants	human	patient-specific iPSCs	*MYH7*	R453C	heterozygous and homozygous genetic editing	dose-dependent cardiomyopathy phenotypes (size, sarcomeric disarray, altered calcium handling)	[104]
promoter	human	IHKE cells	*DSG2*	rare variant	risk and non-risk alleles *in vitro*	reduced DSG2 transcriptional activity associated with minor allele preferential AP-1 binding to non-risk allele	[122]
***obesity***							
distal enhancer	human mouse	brain tissue whole organism	*FTO/IRX3*	rs9930506	IRX3 knock-out	robust interaction between intronic FTO variants and IRX3 promoter in brain tissue IRX3-deficient mice show phenotypes consistent with role in control of body mass	[123]

### 4.1. Disruption of transcription factor binding sites

Genetic variants associated with CVD have long been postulated to result in altered binding of transcription factors, such as those directing cardiac development. Pioneering work by Smemo *et al*. [30] integrated functional genomics and targeted sequencing of CHD patients to identify a mutation within an enhancer element for the *TBX5* gene segregating with the disease. *In silico* analyses suggested the mutation alters a binding site for the TAL1 transcription factor, and disrupted the activity of the enhancer *in vitro* and in transgenic mouse and zebrafish models. Similarly, Kapoor *et al.* [116] carried out a detailed mapping of GWAS variants associated with LQTS in the *NOS1AP* locus, including integration with available functional genomics data. By interrogating 12 variants in reporter assays, the authors revealed a genetic variant in an enhancer element proximal to *NOS1AP*. This SNP clearly affected enhancer activity *in vitro*, although the identity of the bound protein remains unknown.

More recently, non-coding variants associated with AF in the *PITX2c* locus have been analysed using a diverse experimental toolkit [59] (see also figure 2*c*). This elegant study focused on variant annotation within the LD block most proximal to *PITX2c*, out of four showing statistical association in this locus. Through the integration of genomic data and reporter assays, the authors identified a potentially functional enhancer variant. Generation of a CRISPR/Cas9 model of the risk allele in hESC-derived CMs showed a 29% decrease in enhancer RNAs from this region, suggesting the risk allele disrupts enhancer activity. The authors combined *in silico* sequence analysis and *in vitro* assays to show the risk variant affects a binding site for the TFAP2A transcription factor which was confirmed through reduced *in vivo* binding of this protein to the risk allele in hESC-CMs. Moreover, downstream effects on *PITX2c* expression were analysed upon deletion of the associated SNP (30–54% reduction), as well as by comparing risk versus non-risk alleles (27% reduction). Lastly, knock-down experiments using interference RNA demonstrated the effects of the regulatory variant being dependent on the expression of TFAP2A and its cofactor CITED2. In a similar vein, a recent functional genomics approach focusing on the analysis of CAD variants in HCASMs after perturbation by pro- or de-differentiation growth factors [109] selected candidate genetic variants based on the integration of epigenetic profiles for chromatin accessibility, enhancer histone marks and transcription factor binding. The authors focused on a candidate intronic variant in the *SMAD3* locus predicted to disrupt a canonical AP1 motif. By combining allele-specific ChIP in HCASMs from individuals heterozygous for this SNP with overexpression and knock-down of AP1 members, this study provided convincing evidence for disruption of AP1 binding by the minor, protective allele—additionally suggesting that, compared to other candidate variants, the greater effect size of this SNP may be due its disruption of an AP1 motif. In sum, these works illustrate the integration of complementary strategies to investigate non-coding genetic variants, and provide compelling mechanistic evidence for their role in disrupting the tissue-specific binding of transcription factors and downstream gene expression (see also table 1 for additional examples).

### 4.2. Long-range gene regulation

As discussed in previous sections, a key challenge in functionally interrogating non-coding genetic variants associated with human disease is the ability of regulatory elements to target genes at very long distances. Although this inherent property of transcriptional enhancers complicates assigning non-coding variants to their likely effector genes, several recent studies provide mechanistic examples for long-range gene regulation mediated by regulatory elements harbouring genetic variants associated with specific forms of CVD, or cardiovascular risk factors such as obesity.

The *SCN5A/SCN10A* locus is genetically associated with congenital heart disease and cardiac arrhythmias such as Brugada syndrome. Van den Boogaard *et al.* [88] reported the first detailed analysis of non-coding variants in this cardiac disease locus, through a combination of functional genomics approaches and reporter assays. Employing the 4C assay for targeted interrogation of three-dimensional chromatin interactions (see §3.2), the authors showed that the most likely functional variant in this locus—which had been ascribed to the *SCN10A* gene based on genomic distance—in fact physically contacted the *SCN5A* promoter, and was associated with allele-specific *SCN5A* expression in humans. In line with studies discussed in the previous section, the risk allele was predicted to disrupt a T-box binding motif, as suggested by *in vitro* reporter assays. Using a similar approach, a recent study characterized variants associated with AF in the *PRRX1* locus [119]. Combining interaction frequency data from published Hi-C studies and locus-specific validation in cardiac fibroblasts, the authors demonstrated a physical interaction between an enhancer element harbouring a disease-associated variant and the *PRRX1* promoter. Moreover, suppression of *PRRX1* expression led to shortening of the atrial action potential in iPSC-derived cardiomyocytes—an electrophysiological hallmark of AF.

Obesity is a major risk factor for CVD, and the genetic basis for its heritability has been the focus of several association studies [124,125]. One of the lead genomic signals associated with obesity in humans corresponds to intronic variants within the *FTO* locus, and follow-up studies have suggested this gene is implicated in the control of body mass [126]. Smemo *et al*. [123] employed a series of elegant experiments to revisit the molecular genetics of this locus. Using 4C-seq to map genomic interactions between the *FTO* intronic region and surrounding promoters, they showed a lack of interaction with the *FTO* promoter in the adult mouse brain. Instead, the authors detected robust interactions between the obesity-associated interval and the *IRX3* gene (located half a megabase downstream). Moreover, obesity-associated SNPs correlated with *IRX3* expression in the human brain, but not with that of *FTO*—suggesting that the association signal overlaps an enhancer element involved in long-range regulation of *IRX3* in the brain. To support this conclusion, the authors performed detailed phenotyping of mice deficient for IRX3, which support a global and hypothalamus-specific role for this gene in control of body mass. Although these results are also compatible with the proposed role of FTO in modulating obesity, they powerfully highlight the importance of long-range gene regulation as a mechanism mediating the effect of genetic variants associated with CVD.

### 4.3. Gene regulation by long non-coding RNAs

A key finding from genomic consortia such as the ENCODE project [81] has been the pervasive transcription of the human genome. Current estimates suggest that up to 80% of our genome may be actively transcribed, including a large variety of non-coding RNA species such as microRNAs, enhancer RNAs and long non-coding RNAs (lncRNAs). In this section, we focus on the emerging roles of lncRNAs in cardiovascular biology (reviewed in [127]), and on recent examples of CVD-associated genetic variants that have been mechanistically linked to lncRNAs.

The potential relevance of lncRNAs on cardiac development was highlighted by the discovery of *FENDRR*, a lncRNA highly expressed in the posterior mesoderm, and whose genetic deletion leads to impaired heart development [128]. Recent work has also implicated lncRNAs in the gene expression response to cardiac stress [129,130]. Focusing on the transcriptional response to pressure overload in mice, Wang *et al.* [131] recently reported and functionally characterized the cardiomyocyte-specific lncRNA *CHAER1*. Employing knock-out mice and perturbation experiments *in vitro*, the authors proposed this lncRNA is necessary and sufficient for cardiomyocyte hypertrophy, which suggested its role in regulating gene expression under cardiac stress. Functional genomics experiments in normal and *CHAER1*-deficient cardiomyocytes indicated negative regulation of H3K27 methylation by *CHAER1*, and further *in vitro* work showed this effect may be mechanistically due to interaction between *CHAER1* and the chromatin repressive protein PRC2—which appears to be transiently enhanced early in response to hypertrophic signals, and precedes a decrease in H3K27me3 and induction of hypertrophic gene expression.

One of the first indications of a putative role of lncRNAs in heart disease came from genetic studies identifying a susceptibility locus for MI [132], encoding a lncRNA termed MI associated transcript (*MIAT*). However, transcriptomic studies of mouse brain development suggest *MIAT* may have pleiotropic effects on gene expression, including on cardiovascular-relevant pathways such as WNT signalling [133]. In addition, this lncRNA has also been linked to fibrosis in HCM and disease prognosis [134]. More recently, detailed experimental annotation of the *KNCH2* locus (associated with QT interval duration in humans) identified regulatory elements in the mouse controlling expression of its two isoforms *KCNH2A* and *B*, one of which controls a lncRNA likely involved in *KCNH2* regulation [98]. Lastly, genetic association studies of CAD independently identified the 9p21 susceptibility locus, which is adjacent to the lncRNA *CDKN2B-AS1* (also known as ANRIL), and hundreds of kilobases from the protein-coding genes *CDKN2A* and *CDKN2B*. Although the biological function of ANRIL and the nearby disease-associated variants is incompletely understood, recent studies have provided evidence for its role in CVD [101,106,107,112]—mechanistic insights into such complex genetic loci will be the focus of the next section.

### 4.4. Mechanistic insights on complex genetic loci

The variety of genetic, epigenomic and molecular approaches currently available for the study of non-coding genetic variants is beginning to allow very detailed investigation of complex genetic loci, such as those harbouring multiple susceptibility variants, or associated with diverse cardiovascular phenotypes. In this section, we discuss two recent studies that exemplify the power of integrative experimental approaches to provide mechanistic insights into complex disease genetics.

Gupta and colleagues [110] recently conducted a detailed study of the 6p24 cardiovascular risk locus, overlapping the *PHACTR1* gene and associated with susceptibility to several CVDs, such as CAD, migraine headache and hypertension. Using imputation-based fine mapping and probabilistic estimation of causal SNPs in the locus, the authors focused on a lead variant showing association of its minor allele with increased risk for CAD, but decreased risk of migraine headache or hypertension—suggesting a role in endothelial function. The study next employed available epigenomic datasets to show the likely causal SNP overlaps an enhancer element with strong H3K27ac signal specifically in aortic tissue. Using an *in vitro* system of iPSC-derived endothelial and vascular smooth muscle cells, the authors conducted elegant gene editing experiments for biallelic deletion of an approximately 90 bp region containing the enhancer variant, which led to higher expression of the distal endothelin-1 (*EDN1*) gene. These results were combined with similar experiments comparing iPSC clones edited to harbour homozygous major (A/A) and homozygous minor (G/G) versions of the lead SNP. Gene expression profiling of these clones showed over 200 differentially expressed genes, which included known regulators of vascular function (e.g. *COL4A1* or *FN1*). Among differentially expressed genes, *EDN1* was the closest to the lead variant, with the minor allele driving an approximately 20% increase in *EDN1* expression. Although clearly consistent with the enhancer variant acting through the regulation of *EDN1*, chromatin-conformation capture experiments failed to show a robust interaction between this variant and the *EDN1* promoter.

Based on these observations, the authors proposed a model where the differing associations of the 6p24 locus with risk for CVDs can be explained by context-dependent effects of EDN1 on the vasculature. First, increased endothelial production of EDN1 (associated with the risk allele) and binding to the ET_A_ receptor can promote atherosclerosis. Second, increased EDN1 binding to the same receptor in VSMCs leads to vasoconstriction (and reduced susceptibility to migraine). Lastly, the association of the minor allele with lower systolic blood pressure may be due to EDN1 binding to the ET_B_ receptor in the endothelial cells of large systemic arteries, leading to systemic vasodilation. Overall, these results show the potential of integrating genetic, epigenetic and gene editing approaches for functional investigation of risk variants associated with multiple diseases and phenotypes.

In a similar vein, a recent study focused on the analysis of the 9p21.3 CVD locus [112] (§4.3), an approximately 60 kb region associated with CAD risk, and containing over a hundred genetic variants [11] that commonly occur in one of two haplotypes (‘risk’ and ‘non-risk’). The authors used an elegant set of experiments to functionally analyse the aggregate effect of variants in each haplotype. First, the authors compared previously generated iPSCs homozygous for the non-risk (NN) and risk haplotypes (RR) with homozygous deletion clones of either haplotype. Although these deletions did not seem to impact iPSC function, differentiation of edited clones towards VSMCs showed faster proliferation of mesoderm precursors in RR cells. Deletion of the risk haplotype reverted proliferation rates to those of NN mesodermal progenitors, and resulted in differential expression of over a hundred genes. In fully differentiated VSMCs this effect was amplified, with deletion of the risk haplotype associating with thousands of differentially expressed genes. Deletion of the risk haplotype also seemed to restore VSMCs to a gene expression pattern resembling that of the non-risk haplotype, and accordingly measures of VSMC function (such as contractile force) were reduced in cells harbouring the risk haplotype, and restored to non-risk levels upon deletion of the 60 kb region.

To inform the mechanistic basis of the gene expression and functional changes associated with the risk haplotype, the authors focused on the complex splicing of the *CDKN2B-AS1* lncRNA (the terminal exons of which are contained in the risk interval). Short isoforms were specifically detected in VSMCs harbouring the risk haplotype, and their overexpression in non-risk VSMCs induced morphological and functional changes resembling those in RR VSMCs, such as reduced contractile force. However, gene expression changes upon expression of short *CDKN2B-AS1* isoforms did not recapitulate most of those associated with the risk haplotype—suggesting that the functional effects of risk variants in this locus are not solely mediated by altered expression of *CDKN2B-AS1*. In sum, this study provides a powerful example of combining genetic editing and phenotyping approaches for the detailed characterization of complex disease-associated haplotypes.

## 5. Clinical significance of cardiovascular non-coding variants

The results of cardiovascular GWAS studies (§2) have undoubtedly succeeded in characterizing the genetic architecture of CVDs—especially for prevalent conditions such as CAD and AF, for which large numbers of patients and controls have been analysed. Nevertheless, in terms of clinical applicability, the ultimate goal is to understand cardiovascular pathophysiological mechanisms, establish improved risk prediction methods and develop effective therapies. Functional annotation of GWAS findings (§3) and mechanistic studies of individual loci (§4) have contributed important insights into pathophysiological pathways, yet many challenges remain in leveraging cardiovascular non-coding variation for clinical application.

One area of substantial development is the use of non-coding GWAS signals in risk prediction. Most CVDs are polygenic, and perturbation experiments of individual non-coding variants are consistent with small individual effects on disease susceptibility—which can be aggregated in genetic scores predictive of individual disease risk or treatment response. This strategy has been recently applied to common polygenic diseases, and suggested polygenic risk scores can identify individuals at a similar risk than that conferred by rare monogenic mutations [16]. Because genetic risk scores can be assessed at birth, they may be particularly useful in risk prediction among younger patients with a reduced cumulative impact of lifestyle factors [135]. This caveat may explain why genetic risk scores only modestly improve the cardiology-based prognosis for diseases such as AF [63]. Nevertheless, increased knowledge of the mechanistic significance of GWAS loci will be key in improving risk prediction from non-coding cardiovascular variants.

A second challenge is the substantial missing heritability in current studies. A good example not covered above is blood pressure GWAS (reviewed in [2]). Familial studies of blood pressure estimate up to 50% heritability, yet the collective effect of all GWAS loci only explains approximately 10%. Significant gene–gene and gene–environment interactions, structural variation, rare variants and epigenetic mechanisms likely contribute to this missing heritability. In this regard, resources such as the UK Biobank [136] are instrumental in the development of experimental designs and analytical approaches to systematically evaluate the interaction between disease genetics and the environment. It is also possible that substantial missing heritability remains hidden in GWAS datasets as sub-threshold variants, corresponding to context-dependent risk effects that may be dependent on environmental influences found in particular stages of the disease.

Lastly, the ultimate challenge of CVD genetics is the development of novel treatment strategies based on genetically supported targets [137]. Although much progress is needed in mechanistic understanding of non-coding GWAS variants, these already point to novel therapeutic pathways. First, non-coding variants influence traditional risk factors, such as lipid metabolism, that may be treatment targets themselves. An example is the *PCSK9* CAD risk locus. PCSK9 functions in LDL cholesterol metabolism and several compounds are in development to inhibit its effect in the circulation. Second, non-coding GWAS variants are also key in suggesting disease-driving molecular processes that could be exploited therapeutically, such as an altered atrial gene regulatory network in AF. Lastly, non-coding genetic variation can also be used to develop individualized treatment strategies. Although the success of genetic testing for patient stratification has been limited to date, individuals with high genetic risk scores appear to have distinct therapeutic responses, such as a larger benefit from statin treatment [138]. Nevertheless, the clinical application of polygenic risk scores for therapeutic targeting is currently hampered by relatively low precision [139].

## 6. Conclusion and future perspectives

Facilitated by key technological developments in sequencing technology, genetic editing and integrative analyses, CVD genetics has made tremendous progress in identifying and prioritizing human genomic intervals associated with disease predisposition. Genetic association studies now involve global efforts to comprehensively characterize putative susceptibility loci. Critically, complementary experimental approaches allow very detailed epigenetic annotation of disease-associated loci, and are providing important insights into the genomic and molecular complexity of gene regulatory programmes in disease-relevant contexts. Lastly, integrative strategies leveraging multi-level approaches are beginning to inform the role of genetic variants and risk haplotypes in cellular and complex phenotypes.

This progress notwithstanding, a number of key challenges remain. First, mechanistic insights for the vast majority of loci associated with CVD are yet to be determined, posing a daunting task that calls for novel strategies to prioritize loci for functional testing. Second, many disease-associated regions harbour complex haplotypes with dozens to hundreds of variants, and determining the contribution of individual SNPs in complex genetic loci will likely require new experimental approaches. Third, most CVDs with a genetic component also occur in the context of multiple environmental factors, such as metabolic disease and chronic cardiac stress. Untangling the contributions of genetic and environmental cues to complex CVD constitutes a primary challenge in the field that are likely to require improved integration of genetic findings and experimental modelling of environmental risk factors.

A number of emerging approaches and models have begun to address these. Data integration strategies incorporating multiple experimental datasets and genetic GWAS findings are increasingly being used to rank non-coding genetic variants for functional experiments [8,102]. Further development of *in vitro* modelling of subtype-specific CVD [140] will be needed to fully realize the potential of human ‘disease in a dish’ studies. The complex nature of many CVDs is also calling for improved strategies to evaluate tissue-specific contributions experimentally, such as the development of engineered human tissues [141]. Lastly, novel high-throughput approaches to screen gene expression contributions [105] and tissue-specificity [142] of regulatory elements (and the non-coding variants within) should be pivotal in gleaning mechanistic insights into how gene regulation contributes to onset and progression of CVDs.

## Data accessibility

This article has no additional data.
